# Effects of Cultivation Substrate Differences on Quality Formation and Polysaccharide Composition Characteristics of *Tremella fuciformis*

**DOI:** 10.3390/jof12040261

**Published:** 2026-04-03

**Authors:** Jianqiu Chen, Yating Deng, Yujie Chen, Keming Zhu, Xun Yao, Shenqiao Yang, Liding Chen, Shujing Sun

**Affiliations:** 1 College of Life Sciences, Fujian Agriculture and Forestry University, Fuzhou 350002, Chinazkmssgwd@163.com (K.Z.);; 2 Gutian Edible Fungi Research Institute, Fujian Agriculture and Forestry University, Ningde 352200, China; 3 Fujian Edible Fungi Industry Technology Innovation Research Institute, Fuzhou 350002, China

**Keywords:** *T. fuciformis*, cultivation substrate, quality, untargeted metabolomics, monosaccharide composition

## Abstract

Cultivation substrate critically affects the quality of *Tremella fuciformis*. Five substrates, including cottonseed hulls (MZKs), *Machilus pauhoi* Kanehira sawdust (BNM), lotus seed hulls (LZKs), *Corethrodendron scoparium* sawdust (HB), and palm fiber (ZL), were evaluated for their effects on agronomic traits, nutritional composition, texture, and taste characteristics. Untargeted metabolomics was applied to elucidate substrate-associated metabolic variations, and polysaccharide monosaccharide composition was quantitatively analyzed. The results showed that the BNM group exhibited the highest fresh weight, whereas the LZK group presented the highest dry weight and crude polysaccharide content. The ZL group displayed the greatest ear piece thickness and fruiting body elevation. Higher protein contents were observed in the ZL and LZK groups, with no differences in crude fiber content. Texture analysis indicated that hardness was highest in the LZK group, whereas the MZK group showed better springiness, cohesiveness, and chewiness. Regarding taste characteristics, the MZK group exhibited the strongest sweetness, the LZK group showed a markedly higher bitterness, and umami levels were comparable across all groups. Metabolomic analysis revealed that substrate-induced variations in amino acids, saccharides, and taste-related metabolites were significantly associated with nutritional quality and taste attributes of *T. fuciformis*. Polysaccharides of fruiting bodies cultivated on the five substrates consisted of six monosaccharides, with composition ratios similar to those of spore extracellular polysaccharides; among them, differences in glucuronic acid (GlcA) proportion represented a key indicator distinguishing fruiting body polysaccharides from spore polysaccharides. This study revealed the metabolic basis and polysaccharide composition underlying substrate-dependent quality of *T. fuciformis*, supporting substrate optimization for high-quality production.

## 1. Introduction

*Tremella fuciformis* Berk. is an important edible and medicinal fungus whose fruiting bodies are rich in polysaccharides, proteins, and dietary fiber. *T. fuciformis* polysaccharides, the major bioactive constituents, exhibit immune-modulating, antioxidant, and hypoglycemic activities [1,2], and also possess excellent processing and application properties in the food industry [3,4]. The yield and quality of *T. fuciformis* are influenced by various environmental and cultivation factors, including germplasm, light exposure, temperature, humidity, ventilation, and cultivation substrate [5,6]. Among these factors, the cultivation substrate, serving as a medium that directly provides nutrients and physical support, is a key factor influencing the agronomic traits, yield, and metabolic characteristics of the fruiting bodies [6,7,8,9]. However, existing studies have primarily focused on yield and major nutritional components, while systematic investigations of the effects of cultivation substrates on texture, flavor, and metabolite profiles remain relatively limited. Although considerable research has examined the yield and nutritional composition of *T. fuciformis*, comprehensive studies addressing its texture, taste attributes, and underlying metabolic mechanisms remain scarce, thereby constraining a deeper understanding of its quality formation and functional properties.

The contents of proteins, polysaccharides, and crude fiber are key indicators for evaluating the nutritional value of edible fungi [9]. Meanwhile, the quality of edible mushrooms is closely associated with their texture and flavor. Texture is one of the key parameters in sensory evaluation, primarily reflected in the hardness, elasticity, and structural integrity of the fruiting bodies. It not only influences chewing perception but is also closely related to moisture retention capacity, dietary fiber content, and polysaccharide structure [10]. Flavor, on the other hand, is jointly determined by amino acids, organic acids, sugars, and various secondary metabolites [11]. Both texture and flavor play crucial roles in shaping consumer sensory experience and acceptance [12,13]. With advancements in metabolomics, the systematic characterization of metabolic differences in edible fungi under varying cultivation conditions has become feasible, providing an effective approach for elucidating the mechanisms underlying quality formation [9,14,15].

The biological functions of *T. fuciformis* polysaccharides depend not only on content but also on fine structural features, particularly monosaccharide composition, which critically influences physicochemical properties and bioactivity [16]. Variations in monosaccharide types and molar ratios can affect molecular conformation, solubility, viscosity, and receptor interactions, thereby modulating antioxidant and immunoregulatory functions [17,18]. Consequently, the monosaccharide composition of fruiting body polysaccharides reflects biosynthetic pathways and potential functionality, providing a basis for quality evaluation. However, the effects of different substrates or environmental conditions on polysaccharide composition remain unclear.

Based on this, the present study cultivated *T. fuciformis* using MZKs, BNM, LZKs, HB, and ZL as primary substrates. Agronomic traits, production performance, texture, taste properties, and nutritional composition were comprehensively analyzed. Untargeted metabolomics was further applied to elucidate substrate-dependent differences in polysaccharides, protein synthesis, and flavor-related metabolites. In addition, the monosaccharide composition of fruiting body polysaccharides was determined to clarify their compositional characteristics under different substrates. This study aims to reveal the metabolic mechanisms by which cultivation substrates influence the nutritional quality and flavor formation of *T. fuciformis*, providing a scientific basis for substrate selection and optimization for high-quality production.

## 2. Materials and Methods

### 2.1. Materials

“Fuyin Yellow Fungus” (No. TYH-SD1) is a novel *T. fuciformis* strain developed in our laboratory through monosporic hybridization, with parental strains Tr21 and TWW01-AX. Its non-major crop variety identification code is Min. ID 2022007, and it has been deposited in the China Center for Type Culture Collection (CCTCC) under the accession number CCTCC NO: M 2023494. The associated fungus of TYH-SD1, *Annulohypoxylon stygium*, is preserved at Gutian Edible Fungi Research Institute, Fujian Agriculture and Forestry University (Ningde, Fujian, China). BNM was purchased from the Gutian Shunxing Edible Fungi Research Institute (Ningde, Fujian, China), HB from Yuanhui Forestry and Animal Husbandry Co., Ltd. (Alxa League, Inner Mongolia, China), and ZL from Yingdian (Xiamen) Supply Chain Management Co., Ltd. (Xiamen, Fujian, China), while MZKs, LZKs, wheat bran, and gypsum were obtained from Lvhua Edible Fungi Co., Ltd. (Gutian, Ningde, Fujian, China). Complete medium for *T. fuciformis* spores: glucose 15.0 g, peptone 2.0 g, yeast extract 2.0 g, K_2_HPO_4_ 1.0 g, KH_2_PO_4_ 0.46 g, MgSO_4_·7H_2_O 0.5 g, in 1000 mL of water, with pH 7.0. The cultivation formula for *T. fuciformis* is provided in Table S1, with a substrate moisture content of 55–60%. The cultivation was carried out under conditions of 20–25 °C and 80–90% relative humidity for 43 d [13]. The resulting fruiting bodies of *T. fuciformis* are shown in Figure S1.

### 2.2. Determination of Agronomic Traits and Production Performance Indicators

The fresh weight, dry weight, remove pedicle weight, fruiting body elevation, diameter, ear piece thickness, and biological efficiency of *T. fuciformis* were determined as described previously [13]. Biological efficiency was calculated using the following formula: (average fresh weight of a single *T. fuciformis* per cultivation bag/dry weight of substrate per bag) × 100%, where the dry weight of substrate per stick was 0.68 kg.

### 2.3. Determination of Nutritional Components

The crude polysaccharide content of *T. fuciformis* fruiting bodies was determined using the phenol–sulfuric acid method [19]. The protein and crude fiber contents of the fruiting bodies were measured according to previously reported methods [20].

### 2.4. Determination of Textural Properties

Textural properties were measured using a TMS-Touch texture analyzer (FTC, Sterling, VA, USA) [13]. Four uniformly sized ear pieces were selected from each *T. fuciformis* fruiting body to determine five parameters: hardness, cohesiveness, springiness, adhesiveness, and chewiness. Each sample was analyzed in triplicate. The test conditions were as follows: TPA-10N program, 6 mm cylindrical probe, target distance of 10 mm, trigger force of 0.50 N, deformation force of 40%, and test speed of 60 mm/min.

### 2.5. Determination of Taste Characteristics

Fresh *T. fuciformis* samples were homogenized and extracted in boiling water for 5 min at a material-to-liquid ratio of 1:40. The extract was centrifuged at 3000 r·min^−1^ for 10 min, and the supernatant was collected. An electronic tongue (iTongue30, THINKSENSO & SENSO, Zheke Instrument Equipment Co., Ltd., Hangzhou, Zhejiang Province, China) was used to evaluate five taste attributes of the samples: sourness, bitterness, saltiness, sweetness, and umami.

### 2.6. Untargeted Metabolomic Analysis

Fruiting bodies with vigorous growth and uniform morphology were selected as samples, with three biological replicates per treatment. The samples were vacuum freeze-dried for 63 h. Approximately 30 mg of the freeze-dried powder was accurately weighed and extracted with 1.5 mL of prechilled (−20 °C) 70% methanol containing internal standards. The mixture was thoroughly vortexed intermittently for extraction, followed by centrifugation at 12,000 r·min^−1^ for 3 min. The supernatant was filtered through a 0.22 μm membrane and transferred into vials for UPLC–MS/MS analysis. The internal standard solution was prepared by diluting a 1000 μg/mL stock solution of the standard compound to a working concentration of 250 μg/mL.

Sample analysis was performed using a Waters ACQUITY UPLC system equipped with an HSS T3 column (1.8 µm, 2.1 mm × 100 mm) (Waters Corporation, Milford, MA, USA). The mobile phase consisted of (A) ultrapure water containing 0.1% formic acid and (B) acetonitrile containing 0.1% formic acid. The column temperature was maintained at 40 °C, with a flow rate of 0.40 mL/min and an injection volume of 4 µL. The gradient elution program was as follows: 0–2.0 min, 95–75% A; 2.0–4.0 min, 75–1% A; 4.0–4.5 min, hold at 1% A; 4.5–4.6 min, return to 95% A; and maintain until 6.0 min.

Mass spectrometric detection was conducted using a Q Exactive HF-X mass spectrometer (Thermo Fisher Scientific, Waltham, MA, USA) operated in both positive (ESI^+^) and negative (ESI^−^) ionization modes, with spray voltages of 3500 V and 3200 V, respectively. The sheath and auxiliary gas flow rates were set to 30 and 5 Arb, respectively. The ion transfer tube temperature was 320 °C, and the vaporizer temperature was 300 °C. The scan range for both MS^1^ and MS^2^ was *m*/*z* 84–1250, with resolutions of 35,000 and 17,500, respectively. The automatic gain control (AGC) target values were 1.0 × 10^6^ and 2.0 × 10^5^, and collision energies of 30, 40, and 50 eV were applied. The dynamic exclusion time was set to 3 s.

Principal component analysis (PCA) and orthogonal partial least squares discriminant analysis (OPLS-DA) were employed to reveal metabolic differences among groups. Differential metabolites were screened according to two criteria: variable importance in projection (VIP) value > 1.00 in the first principal component of the OPLS-DA model, and Student’s *t*-test *p* < 0.05. Metabolic pathways were annotated and analyzed based on the KEGG database (http://www.genome.jp/kegg/pathway.html, accessed on 10 October 2025) [15].

### 2.7. Determination of Monosaccharide Composition and Chemometric Analysis of T. fuciformis Fruiting Body Polysaccharides

The preparation of fruiting body polysaccharides and the analysis of their monosaccharide composition were performed following the method previously reported by Chen et al. [16]. Chemometric analysis was conducted by integrating the monosaccharide composition data of polysaccharides from TYH-SD1 spores produced from Chen et al. [16].

### 2.8. Determination of Intracellular and Extracellular Polysaccharide Yields of T. fuciformis Spores

Mature, intact, and uncontaminated *T. fuciformis* fruiting bodies were selected, and tissue blocks of 1–2 mm were excised from the central pileus under a laminar flow hood. The blocks were first immersed in 75% ethanol for 1 min, followed by 2–3 rinses with sterile water. The tissue blocks were then inoculated onto PDA plates and incubated upside down at 23 °C for 5–10 d. Once yeast-like spores were observed, purification was performed using streaking or spreading methods. The purified spores were inoculated onto PDA medium and incubated with shaking for 7 d, following the method of Chen et al. [16], to determine intracellular and extracellular polysaccharide contents.

### 2.9. Data Statistics and Analysis

Significance analysis was performed using IBM SPSS Statistics 26 software (IBM Corp., Armonk, NY, USA). Figures were generated using Origin 2024 (OriginLab Corp., Northampton, MA, USA) and the Metware Cloud platform (https://cloud.metware.cn/, accessed on 15 October 2025). All experiments were conducted with at least three replicates.

## 3. Results

### 3.1. Analysis of Agronomic Traits and Production Performance

The effects of different cultivation substrates on the agronomic traits and production performance of *T. fuciformis* are shown in Table 1. The fresh weight per fruiting body in the BNM group reached 123.33 g, which was significantly higher than that of the other groups (*p* < 0.05). Meanwhile, the dry weight in the LZK group reached 35.47 g, significantly higher than that in other groups (*p* < 0.05). The BNM group showed the highest biological efficiency (54.41%), followed by the LZK group (48.86%). The ZL group displayed favorable agronomic traits, including an ear piece thickness of 0.20 mm, an elevation of 51.93 mm, and a diameter of 120.42 mm. Overall, these results indicated that BNM was more suitable for increasing fresh yield, LZKs promoted dry matter accumulation, and ZL contributed to superior commercial traits of *T. fuciformis*.

### 3.2. Quality Analysis

#### 3.2.1. Analysis of Nutritional Components

**Figure 1:**
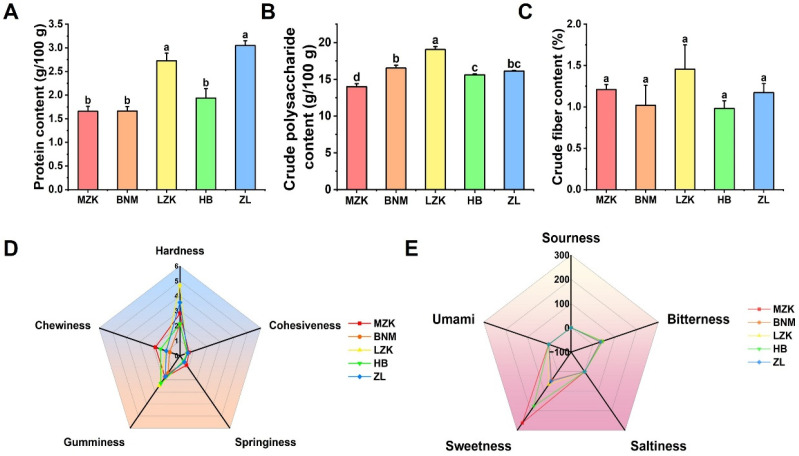
Effects of different cultivation substrates on the qualities of *T. fuciformis.* (**A**) Protein, (**B**) crude polysaccharides, (**C**) crude fiber, (**D**) textural properties, (**E**) taste characteristics. Different lowercase letters indicate significant differences at *p* < 0.05; *n* = 3 for each group.

The protein, crude polysaccharide, and crude fiber contents of *T. fuciformis* cultivated on different substrates are shown in Figure 1A–C. the ZL group exhibited the highest protein content (3.05 g/100 g), followed by the LZK group (2.73 g/100 g). The LZK group also showed the highest crude polysaccharide content (19.06 g/100 g, *p* < 0.05), followed by the BNM and ZL groups, with values of 16.54 g/100 g and 16.12 g/100 g, respectively. The MZK group had the lowest crude polysaccharide content, at only 14.00 g/100 g. No significant differences were observed in crude fiber content among the cultivation groups. These results indicated that LZKs as a cultivation substrate promote polysaccharide synthesis in *T. fuciformis*, effectively enhancing their nutritional value, while crude fiber content was minimally affected by the cultivation substrate.

#### 3.2.2. Texture Analysis

Different cultivation substrates significantly influence the textural properties of *T. fuciformis* fruiting bodies. As shown in Figure 1D, the MZK group exhibited the highest cohesiveness, springiness, and chewiness, indicating a more resilient texture and superior tensile strength. The LZK group exhibited significantly higher hardness than other experimental groups, but relatively lower springiness and chewiness, indicating that the ear pieces were easier to chew and more compact, which is beneficial for storage and transportation. The BNM and HB groups exhibited relatively lower values across all texture parameters, with reduced hardness, springiness, and cohesiveness, resulting in a comparatively loose tissue structure. In contrast, the ZL group displayed intermediate levels of hardness, springiness, and cohesiveness, with balanced texture parameters and a dense, stable tissue structure.

#### 3.2.3. Analysis of Taste Characteristics

The cultivation substrate had a significant effect on the taste characteristics of *T. fuciformis* (Figure 1E). Sourness was significantly higher in the MZK, BNM, and HB groups compared to the LZK and ZL groups (*p* < 0.05). In terms of bitterness, the MZK, LZK, and HB groups showed elevated levels, significantly higher than those in the BNM and ZL groups (*p* < 0.05). Salty taste was highest in the HB group, significantly exceeding the other groups (*p* < 0.05), followed by the BNM group, while the MZK, LZK, and ZL groups exhibited lower levels. Sweetness showed the greatest variation, with the MZK group reaching the highest level, followed by the HB group. The LZK, BNM, and ZL groups were significantly lower (*p* < 0.05), with the BNM group exhibiting the lowest sweetness. Umami taste did not differ significantly among the groups.

### 3.3. Metabolomic Analysis of T. fuciformis Cultivated on Different Substrates

#### 3.3.1. Analysis of Metabolite Composition

**Figure 2:**
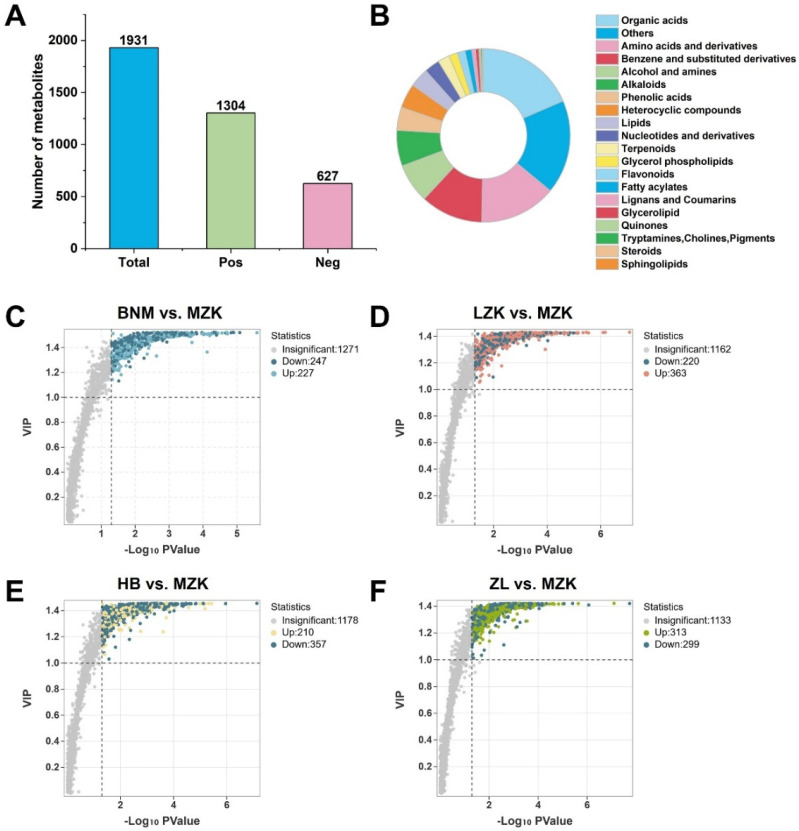
Metabolite composition and volcano plots of differential metabolites of *T. fuciformis* cultivated on different substrates. (**A**) Overall metabolite composition, (**B**) metabolite composition by primary classification, (**C**–**F**) volcano plots of differential metabolites.

Untargeted metabolomics was performed using liquid chromatography–tandem mass spectrometry (LC-MS/MS). A total of 1931 identifiable metabolites were detected, of which 1304 were identified in positive ion mode and 627 in negative ion mode (Figure 2A). After merging identical metabolites from both ionization modes, 1745 metabolites were confirmed. Based on primary classification, these metabolites were grouped into 20 categories. Organic acids were the most abundant, comprising 372 metabolites, accounting for 19.3%, followed by amino acids and their derivatives with 274 metabolites (14.2%), and alcohols and amines totaling 132 metabolites (6.8%) (Figure 2B). Among the top 20 metabolites ranked by relative abundance, 17 were common across samples, though their abundance levels varied (Figure S2). These results indicated that while the metabolic processes of *T. fuciformis* exhibit certain commonalities, differences in substrate absorption and utilization lead to variations in metabolic activity and metabolite accumulation.

(1) *PCA*

PCA indicated that the first principal component (PC1) and the second principal component (PC2) explained 24.4% and 16.2% of the variance among samples, respectively, accounting for a total of 40.6% of the overall variance (Figure S3). These results suggested that different cultivation substrates have a pronounced effect on the metabolite composition of *T. fuciformis*.

(2) *OPLS-DA analysis*

Using MZKs as the control group, OPLS-DA was employed to compare metabolic differences among other groups of *T. fuciformis*. All model parameters demonstrated excellent performance (R^2^X ≥ 0.597, R^2^Y = 1, Q^2^ ≥ 0.893) (Figure S4), indicating that this model possessed high fitting accuracy and predictive capability, making it suitable for subsequent analyses.

(3) *Statistics of differential metabolites*

Differential metabolites were selected using OPLS-DA with VIP ≥ 1 and *p*-value < 0.05, identifying significant differences among multiple groups (Figure 2C–F). Compared to the MZK group, the BNM, LZK, HB, and ZL groups exhibited 474 (227 upregulated, 247 downregulated), 583 (363 upregulated, 220 downregulated), 567 (210 upregulated, 357 downregulated), and 612 (313 upregulated, 299 downregulated) differential metabolites, respectively. These results indicated that different cultivation substrates have a significant impact on the metabolic regulation of *T. fuciformis*.

(4) *Analysis of differential amino acids and derivatives*

**Figure 3:**
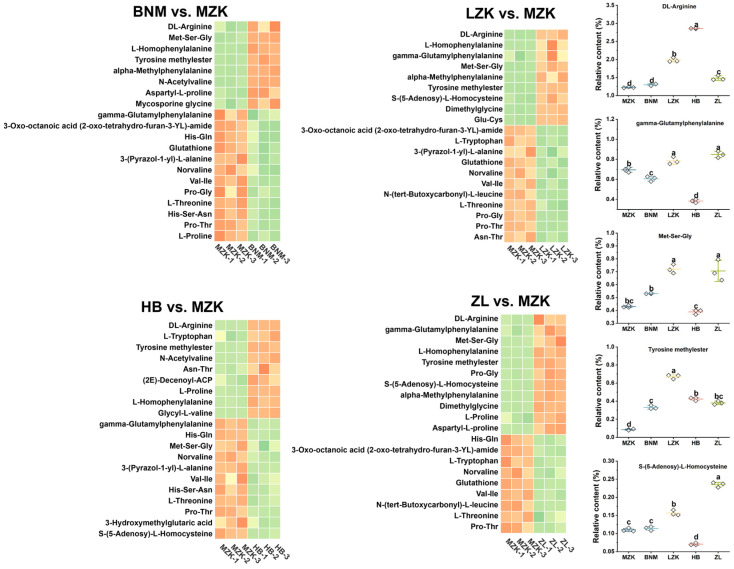
Distribution of the top 20 differential amino acids and derivatives in *T. fuciformis* fruiting bodies cultivated on different substrates. Different lowercase letters indicate significant differences at *p* < 0.05.

The total relative content of amino acids and their derivatives in the LZK, HB, and ZL groups were 16.12%, 14.99%, and 15.07%, respectively, whereas the MZK and BNM groups exhibited lower levels (Figure S5A), consistent with the measured protein content (Figure 1). The distribution of the top 20 differential amino acids and derivatives across different substrates is shown in Figure 3. In the LZK and ZL groups, the levels of γ-glutamylphenylalanine, methionyl-seryl-glycine (Met-Ser-Gly), S-(5-adenosyl)-L-homocysteine, and tyrosine methyl ester were significantly higher than in the MZK control. These metabolites need to be broken down to generate their corresponding amino acid monomers, which can then be used for protein synthesis [21,22,23,24]. This suggested that the amino acid precursors available for direct protein synthesis in the LZK and ZL groups may have been largely consumed, reflecting more active protein synthesis metabolism in these groups. DL-arginine exhibited the highest relative abundance among the five groups, exceeding 1.22% in all cases, with the HB group showing the highest level and the MZK and BNM groups the lowest. DL-arginine is an important substrate for protein synthesis and a common flavor-contributing amino acid [25,26].

(5) *Analysis of differential sugar metabolites*

Saccharide metabolites include a variety of precursors used for polysaccharide synthesis [27,28]. The relative abundances of sugar metabolites among the different groups did not differ significantly, indicating that overall sugar metabolism was relatively consistent (Figure S5B). Analysis of differential sugar metabolites revealed that the four comparison groups contained 14 (BNM vs. MZK), 21 (LZK vs. MZK), 20 (HB vs. MZK), and 20 (ZL vs. MZK) differential metabolites, respectively. In the BNM vs. MZK group, eight metabolites were upregulated and six were downregulated; in the LZK vs. MZK group, 13 were upregulated and eight were downregulated; and both the HB vs. MZK and ZL vs. MZK groups contained 12 upregulated and eight downregulated metabolites (Figure S5E).

**Figure 4:**
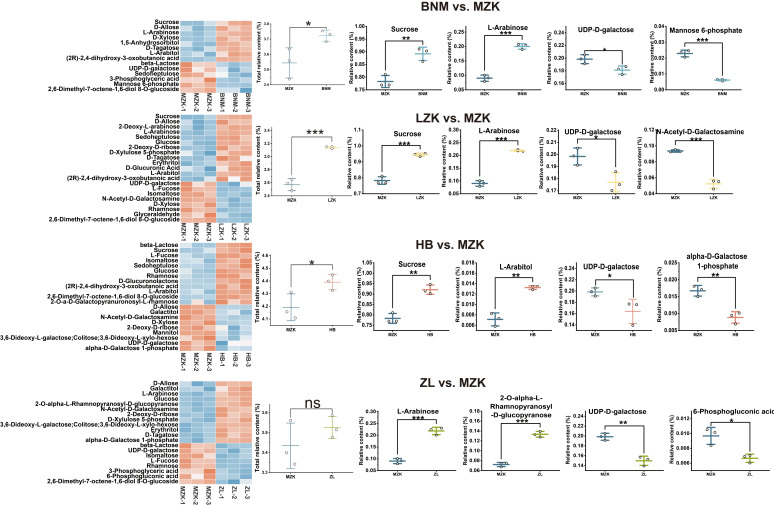
Distribution of differential sugar metabolites in *T. fuciformis* fruiting bodies cultivated on different substrates. *, **, and *** indicate significant differences at *p* < 0.05, *p* < 0.01, and *p* < 0.001, respectively.

The distribution of differential carbohydrate metabolites is presented in Figure 4. With the exception of the ZL vs. MZK comparison, the LZK, BNM, and HB groups exhibited significantly higher levels of differential carbohydrate metabolites compared to the control group (MZK). Notably, sucrose, L-arabinose, and derivatives, L-arabitol and 2-O-α-L-rhamnopyranosyl-D-glucopyranose, showed relatively elevated abundances, whereas metabolites such as UDP-D-galactose, mannose 6-phosphate, and α-D-galactose 1-phosphate were more abundant in MZK group.

Both sucrose and 2-O-α-L-rhamnopyranosyl-D-glucopyranose are disaccharides and cannot be directly incorporated into *T. fuciformis* polysaccharide synthesis. Previous studies indicate that L-arabinose is absent in *T. fuciformis* polysaccharides [16]; thus, its presence may reflect accumulation or conversion into other reaction products. This may explain why the LZK, BNM, and HB groups, which exhibited higher polysaccharide content than the control, accumulated more of these sugars. Additionally, the experimental groups may demonstrate greater conversion efficiency of other monosaccharides that can enter polysaccharide biosynthetic pathways, such as mannose and glucuronic acid, preventing their accumulation.

UDP-D-galactose, mannose 6-phosphate, and α-D-galactose 1-phosphate are metabolites in the polysaccharide biosynthesis pathway, while 6-phosphogluconic acid and N-acetyl-D-galactosamine are derivatives of these pathway metabolites [29,30]. The accumulation of these compounds in the MZK group suggested that *T. fuciformis* in this group has lower conversion efficiency for metabolites in the polysaccharide biosynthetic pathway, thereby limiting overall polysaccharide synthesis.

(6) *Analysis of differential taste-related metabolites*

The primary taste-contributing components of edible fungi include non-volatile substances, such as free amino acids, soluble sugars, and taste-active nucleotides, as well as volatile compounds. Among these, sulfur-containing compounds and alcohols/ketones impart the characteristic mushroom flavor, while aldehydes and esters contribute delicate aromatic notes, collectively forming the complex and unique flavor profile of edible fungi [11]. The levels of taste-active compounds in the MZK group of *T. fuciformis* were significantly higher than those in the LZK and ZL groups (Figure S5C), indicating a richer flavor profile in the MZK group. Statistical analysis of the top 20 differential taste-related metabolites showed that the four comparison groups contained 124 (BNM vs. MZK), 153 (LZK vs. MZK), 154 (HB vs. MZK), and 162 (ZL vs. MZK) differential metabolites, respectively. Specifically, the BNM vs. MZK group included 63 upregulated and 61 downregulated metabolites; the LZK vs. MZK group included 60 upregulated and 93 downregulated metabolites; the HB vs. MZK group included 88 upregulated and 66 downregulated metabolites; and the ZL vs. MZK group included 82 upregulated and 80 downregulated metabolites (Figure S5F).

**Figure 5:**
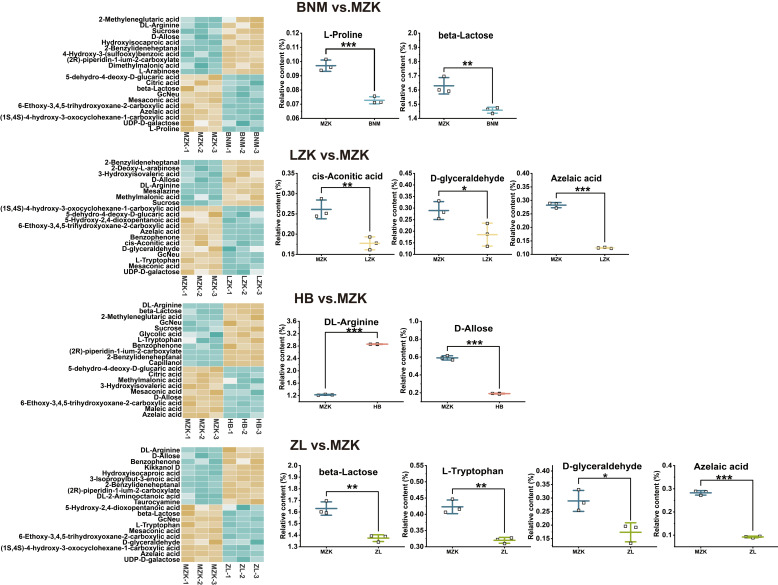
Distribution of the top 20 differential taste-related metabolites in *T. fuciformis* fruiting bodies cultivated on different substrates. *, **, and *** indicate statistical significance at *p* < 0.05, *p* < 0.01, and *p* < 0.001, respectively.

The distribution of taste-differentiating metabolites is shown in Figure 5. The MZK group exhibited higher levels of azelaic acid and cis-aconitic acid in *T. fuciformis*, corresponding to the significantly enhanced sour taste in this group [31]. The relative content of L-proline and L-tryptophan increased, consistent with the stronger bitter taste observed [32]. Additionally, the MZK group was rich in beta-lactose, D-allose, and the non-sugar sweetener D-glyceraldehyde, metabolites that significantly contribute to sweetness [33,34,35]. In contrast, the HB group *T. fuciformis* exhibited higher DL-arginine content, potentially enhancing salty taste perception [36].

#### 3.3.2. KEGG Enrichment Analysis

The results of KEGG enrichment analysis for differential metabolites were presented in Figure S6. In all four comparison groups, two metabolic pathways—biosynthesis of amino acids and amino sugar and nucleotide sugar metabolism—were consistently enriched.

**Figure 6:**
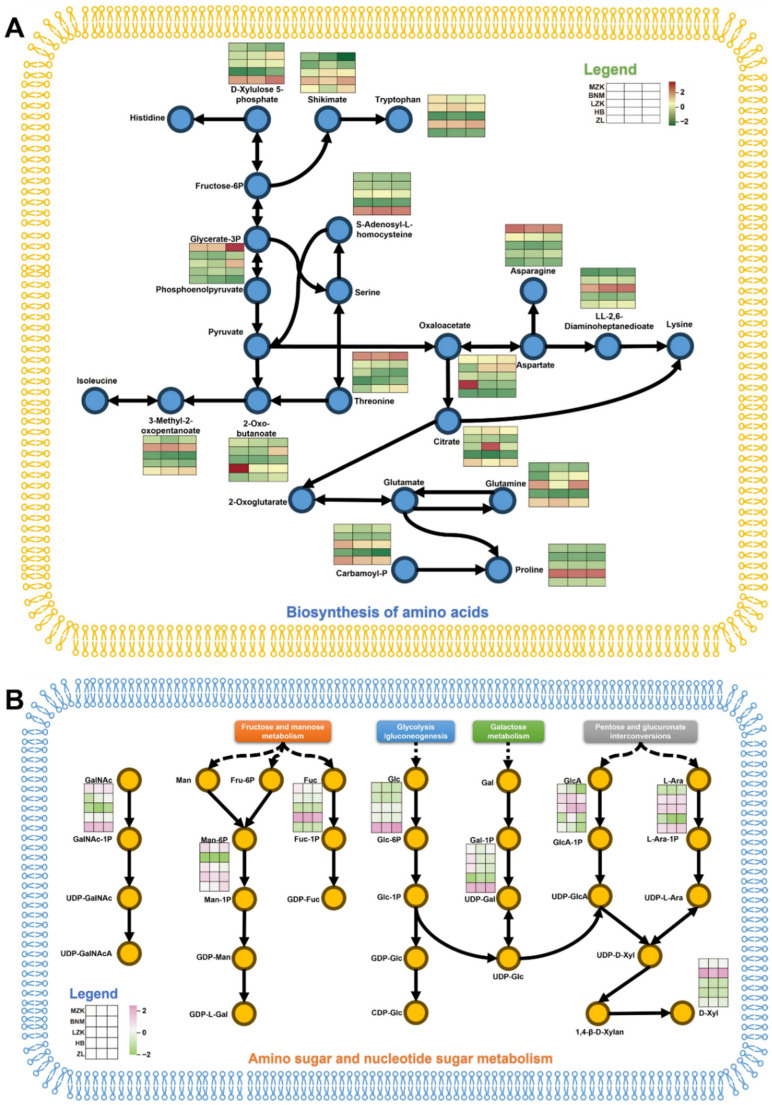
Analysis of key KEGG metabolic pathways. (**A**) Biosynthesis of amino acids, (**B**) amino sugar and nucleotide sugar metabolism.

The amino acid biosynthesis pathway detected 15 differentially expressed metabolites (Figure 6A). The LZK and ZL groups exhibited significantly higher levels of carbamoyl phosphate and S-(5-adenosyl)-L-homocysteine compared to other groups. Both are key intermediates in amino acid synthesis that require further conversion to generate amino acids usable for protein synthesis. This result was consistent with the analysis of differential amino acids and derivatives (Figure 3).

The amino sugar and nucleotide sugar metabolism pathway provides key activated monosaccharide precursors for polysaccharide biosynthesis, such as UDP-Glc, UDP-Gal, and GDP-Man [29,30]. Under the catalysis of glycosyltransferases, these precursors are sequentially linked to form complex polysaccharide chains, representing a central step in fungal cell wall construction, polysaccharide synthesis, and glycoconjugate assembly [30,37]. Within this pathway, nine differential metabolites were detected; however, in the LZK group, which exhibited the highest crude polysaccharide content, no significantly enriched metabolites were observed (Figure 6B). This finding may reflect a more efficient polysaccharide biosynthesis process, whereby intermediate metabolites are rapidly converted into end products for polysaccharide assembly, resulting in lower accumulation of pathway metabolites. These results further suggested that the lotus seed shell substrate facilitates efficient synthesis and accumulation of polysaccharides in *T. fuciformis*.

### 3.4. Monosaccharide Composition Analysis of Fruiting Body Polysaccharides

**Figure 7:**
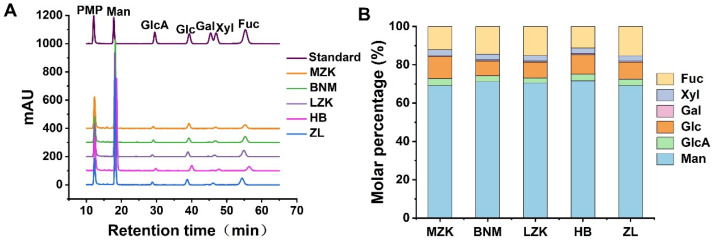
Monosaccharide composition of polysaccharides from *T. fuciformis* fruiting bodies cultivated on different substrates. (**A**) HPLC monosaccharide profiles, (**B**) molar percentage of individual monosaccharides. *n* = 3 for each group.

The HPLC profiles of monosaccharide composition in fruiting body polysaccharides from *T. fuciformis* cultivated on different substrates are shown in Figure 7A. All polysaccharides were composed of six monosaccharides: mannose (Man), glucuronic acid (GlcA), glucose (Glc), galactose (Gal), xylose (Xyl), and fucose (Fuc), consistent with previous reports [16]. As shown in Figure 7B, among the five substrate formulations, Man exhibited the highest molar percentage, with 69.10 mol% (MZK), 71.10 mol% (BNM), 70.49 mol% (LZK), 71.59 mol% (HB), and 69.10 mol% (ZL). Fuc was the second most abundant, accounting for 12.24 mol% (MZK), 14.44 mol% (BNM), 15.24 mol% (LZK), 11.31 mol% (HB), and 15.28 mol% (ZL). Glc ranked third, with molar percentages of 11.52 mol% (MZK), 7.67 mol% (BNM), 8.15 mol% (LZK), 10.21 mol% (HB), and 8.79 mol% (ZL). In addition, the molar proportions of GlcA and Xyl in the polysaccharides from *T. fuciformis* cultivated under the five formulations were all below 5 mol%, while the molar percentage of Gal was less than 1 mol%.

The molar percentage of Man in fruiting body polysaccharides was significantly higher in the BNM and HB groups compared to the MZK and ZL groups. The MZK group exhibited the highest GlcA molar percentage, and its Glc proportion was significantly higher than that of the other four groups. Among the five substrate formulations, the Gal molar percentage was highest in the BNM group. No significant differences were observed in Xyl proportion across the five groups. The Fuc molar percentage in the BNM, LZK, and ZL groups was significantly higher than in the MZK and HB groups. These results indicated that different cultivation substrates not only influence the overall accumulation of polysaccharides in *T. fuciformis* but also partially alter their monosaccharide composition.

Spores of *T. fuciformis* isolated and purified from fruiting bodies grown under five different substrate formulations were cultivated in shake flasks, and the levels of intracellular and extracellular polysaccharides were comparatively analyzed (Figure S7). No significant differences in polysaccharide yields were observed among treatments. Considering the variations in fruiting body polysaccharide yield and monosaccharide composition under different substrate conditions, it appears that the effect of cultivation substrate may be exerted through regulation of nutrient supply and metabolic allocation during the fruiting body growth stage, rather than through altering the intrinsic genetic traits governing polysaccharide biosynthesis.

### 3.5. Comparative Analysis of Monosaccharide Composition Between Fruiting Body and Spore Polysaccharides

**Figure 8:**
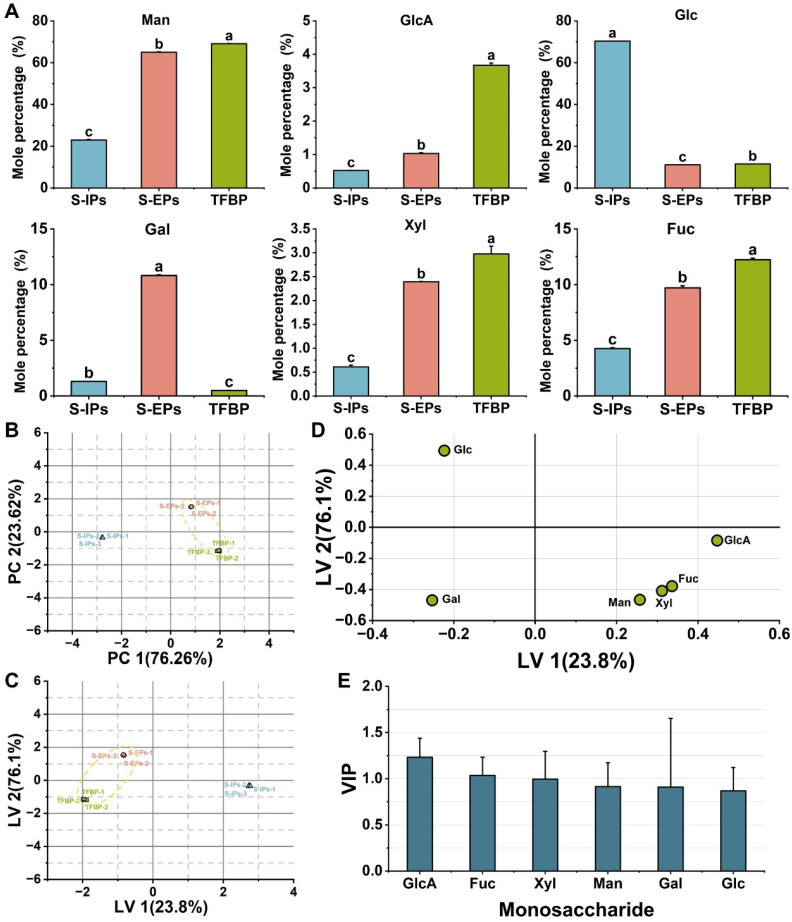
Comparison of monosaccharide composition among intracellular and extracellular polysaccharides from *T. fuciformis* spores [16] and polysaccharides from fruiting bodies. (**A**) Bar chart of molar percentages, (**B**) PCA score plot of HPLC profiles, (**C**) OPLS-DA score plot of HPLC profiles, (**D**) loading plot, (**E**) variable importance in projection (VIP) plot. Different lowercase letters indicate significant differences at *p* < 0.05; *n* = 3 for each group.

The monosaccharide composition of fruiting body polysaccharides (TFBPs) was compared with previously reported polysaccharides (S-EPs and S-IPs) from *T. fuciformis* TYH-SD1 spore (Figure 8A) [16]. The results indicated that TFBPs were more similar to S-EPs, with both dominated by Man. Comparison of the six monosaccharides revealed that the molar percentages of Man, GlcA, Fuc, and Xyl in TFBPs were significantly higher than in S-IPs and S-EPs (*p* < 0.05), whereas Gal was higher in S-EPs, and Glc was higher in S-IPs.

PCA based on the molar percentages of the six monosaccharides showed that the first principal component (PC1, 76.26%) and second principal component (PC2, 23.62%) accounted for 99.88% of the total variance, effectively capturing the variation in monosaccharide composition among samples (Figure 8B). This demonstrated that monosaccharide composition ratios provide strong discriminatory power in polysaccharide structural characterization and classification, serving as a key criterion for distinguishing fruiting body polysaccharides from spore polysaccharides.

OPLS-DA of TFBPs, S-EPs, and S-IPs showed that the major latent variables explained 99.9% of the total variation (Figure 8C), confirming the effectiveness of monosaccharide composition differences in differentiating polysaccharides from different sources. The PCA and OPLS-DA score plots revealed that S-EPs and TFBPs were positioned closely in multivariate space, consistent with their similarity in monosaccharide composition. Variable contribution analysis indicated that GlcA contributed positively to LV1 and Glc positively to LV2, while Man and Gal contributed negatively to LV2, highlighting the importance of these monosaccharides in explaining structural differences among polysaccharides from different sources (Figure 8D). The VIP values of GlcA and Fuc were 1.23 and 1.04, respectively, both exceeding 1 (Figure 8E), suggesting their high discriminative contribution in distinguishing spore and fruiting body polysaccharides [16]. Overall, differences in GlcA content represent a key structural feature for differentiating fruiting body polysaccharides from spore polysaccharides.

## 4. Discussion

In this study, different cultivation substrates influenced both the yield and nutritional composition of *T. fuciformis*. LZKs, a by-product of lotus seeds, are rich in starch (30.4%) [38], which serves as a readily utilizable soluble carbon source for *T. fuciformis*, providing rapid energy supply and participating in metabolic activities. The breakdown of starch generates glucose, which not only fulfills the energy demands for cell growth but also serves as a key monosaccharide substrate for cell wall polysaccharide biosynthesis, thereby contributing to the higher crude polysaccharide content and dry matter accumulation observed in the LZK group [39,40,41]. Moreover, the carbon skeletons derived from starch degradation can support amino acid and protein synthesis via carbohydrate metabolic pathways [42], providing a mechanistic basis for the elevated protein content in the LZK group. The synergistic accumulation of polysaccharides and proteins not only increases dry weight but also enhances the structural firmness of the fruiting bodies, resulting in superior textural hardness of *T. fuciformis* cultivated on the LZK substrate compared to other substrates.

In contrast, MZKs, BNM, HB, and ZL are all by-products of agriculture and wood processing, with compositions similar to common lignocellulosic materials, primarily consisting of cellulose, hemicellulose, and lignin [43,44]. The degradation and conversion of these high-molecular-weight substances were relatively complex and tightly regulated by enzymatic activity, limiting the accumulation of dry matter and nutritional components in *T. fuciformis* compared to the LZK group. No significant differences were observed in crude fiber content among the experimental groups, likely because intracellular metabolic activities associated with fiber synthesis decline once cell morphology is established, reducing the impact of external stimuli on fiber content [45]. This finding is consistent with previous laboratory results measuring crude fiber in *Hypsizygus marmoreus* mycelia and fruiting bodies [9]. Collectively, these results indicate that differences in substrate utilization by *T. fuciformis* may affect the activation of key metabolic pathways and the efficiency of metabolite accumulation, leading to variations in dry matter, crude polysaccharide, and protein levels, which are further reflected in fruiting body texture and flavor characteristics. These findings provide valuable insights for optimizing *T. fuciformis* yield and quality through the modulation of cultivation substrate composition.

Untargeted metabolomics was applied to explore the potential metabolic basis underlying quality formation of *T. fuciformis* under different substrate conditions. In the LZK group, the precursors were extensively utilized for protein and polysaccharide biosynthesis, resulting in minimal accumulation of these readily available precursors, while upregulated complex metabolites largely remained in pre-synthetic transformation stages. By contrast, fruiting bodies grown on other substrates exhibited limited access to high-molecular-weight carbon and nitrogen sources, leading to insufficient energy supply, lower biosynthetic activity for proteins and polysaccharides, and reduced nutrient accumulation. In the MZK group, which showed the lowest crude polysaccharide content, several intermediate metabolites of polysaccharide biosynthesis accumulated, including UDP-D-galactose, D-mannose-6-phosphate, and α-D-galactose-1-phosphate, but were not efficiently incorporated into polysaccharide biosynthesis, suggesting a constraint on polysaccharide production under this substrate. Differences were also observed in the relative contents of taste-related metabolites, including sour, bitter, sweet, and salty compounds, likely reflecting variations in metabolic conversion efficiency across substrates [7]. Collectively, these findings provide important insights into the metabolic responses of *T. fuciformis* to exogenous substrates and the regulatory mechanisms of key metabolic pathways.

Different cultivation substrates exhibited a regulatory effect on the accumulation and composition of *T. fuciformis* polysaccharide; however, no significant differences were observed in polysaccharide synthesis by spores isolated and purified from fruiting bodies. This suggested that substrates, as external factors, may influence polysaccharide biosynthesis through regulation of gene expression and metabolic flux, without altering the genetic traits of the strain [7,46]. These findings indicated that, while maintaining genetic stability, cultivation substrates can modulate nutrient utilization and metabolic allocation, thereby affecting polysaccharide yield and structural characteristics, providing a reference for industrial production of high-polysaccharide *T. fuciformis*.

Monosaccharide composition is widely recognized as a key structural factor influencing polysaccharide charge properties, functional group distribution, and bioactivity, and it serves as one of the most fundamental and representative characterization indices in polysaccharide research [47]. Previous studies have shown that chemometric models based on monosaccharide composition can partially reflect intrinsic structural differences among polysaccharides from spores of different *T. fuciformis* sources [16]. From a structure–function perspective, variations in monosaccharide composition among polysaccharides from different sources may further affect their physicochemical properties and biological behaviors. In particular, GlcA, an acidic monosaccharide containing a carboxyl group, plays a critical role in molecular charge, conformational stability, and intermolecular interactions within polysaccharide systems. In vivo, GlcA participates in detoxification metabolism and serves as a fundamental structural unit of several highly bioactive glycosaminoglycans, such as heparin, chondroitin sulfate, and hyaluronic acid [48]. Consequently, variations in GlcA content are often closely associated with polysaccharide viscoelasticity, biocompatibility, and moisture-retention properties. Studies on *T. fuciformis* polysaccharides suggest that higher GlcA proportions may be linked to enhanced colloidal network formation and moisture-retention capabilities [49,50]. Moreover, polysaccharide structural features are jointly regulated by genetic background and growth environment, and samples obtained from different geographic origins or cultivation conditions may exhibit variations in monosaccharide composition and molar ratios [5]. Against this background, using monosaccharide composition as a relatively simplified and stable structural indicator to compare and classify polysaccharides from different sources is reasonable and informative, providing a foundation for further analysis of functional differences from a structural perspective.

## 5. Conclusions

This study systematically compared, for the first time, *T. fuciformis* cultivated on different substrates in terms of yield, agronomic traits, nutritional quality, texture, and taste attributes. By integrating untargeted metabolomics, the metabolic mechanisms through which substrates influence quality formation were elucidated, and the compositional characteristics and potential variations in polysaccharides under different substrate conditions were clarified. The results showed that substrates modulate the flux and accumulation of metabolites involved in protein, polysaccharide, and flavor compound biosynthesis, leading to differences in nutritional composition and taste. Among the substrates tested, fruiting bodies cultivated on LZKs exhibited the highest overall quality, with superior dry matter accumulation, biological efficiency, textural firmness, and nutrient content, indicating its considerable practical value for cultivation. Polysaccharide analysis indicated that while the fundamental monosaccharide types remained unchanged, the relative proportions were affected, with GlcA proportion differences serving as a key criterion to distinguish fruiting body polysaccharides from spore polysaccharides. No significant changes were observed in polysaccharide content from spores isolated from fruiting bodies, suggesting that substrates primarily influence metabolic flux rather than altering genetic traits. Overall, this study revealed how different cultivation substrates influence the metabolic pathways and polysaccharide composition underlying *T. fuciformis* quality, offering a scientific basis for optimizing substrates to produce high-quality fruiting bodies.

## Figures and Tables

**Table 1:** Agronomic traits and production performance parameters of *T. fuciformis* cultivated on different substrates.

Cultivation Substrates	Main Agronomic Traits and Production Performances						
	Fresh Weight (g)	Dry Weight (g)	Remove Pedicle Weight (g)	Fruiting Body Elevation (mm)	Diameter (mm)	Ear Piece Thickness (mm)	Biological Efficiency (%)
MZK	91.33 ± 4.10 c	24.78 ± 1.33 c	84.43 ± 3.55 c	47.01 ± 5.63 a	114.99 ± 13.09 ab	0.17 ± 0.02 b	40.29 ± 1.41 c
BNM	123.33 ± 15.72 a	27.72 ± 4.58 c	115.57 ± 15.79 a	47.31 ± 7.19 a	121.50 ± 11.58 a	0.18 ± 0.02 ab	54.41 ± 7.08 a
LZK	110.75 ± 13.98 b	35.47 ± 2.88 a	103.23 ± 14.97 b	48.62 ± 3.64 a	108.67 ± 15.29 bc	0.20 ± 0.02 a	48.86 ± 5.74 ab
HB	87.89 ± 4.23 c	21.30 ± 1.62 d	79.54 ± 5.95 c	34.94 ± 6.76 b	101.33 ± 8.35 c	0.17 ± 0.02 b	38.77 ± 1.36 c
ZL	104.08 ± 15.97 b	31.39 ± 2.55 b	96.88 ± 15.92 b	51.93 ± 4.55 a	120.42 ± 6.83 a	0.20 ± 0.03 a	45.92 ± 2.33 bc

Note: Different lowercase letters indicate significant differences at the *p* < 0.05 level; *n* = 3 for each group.

## Data Availability

The original contributions presented in this study are included in the article/Supplementary Materials. Further inquiries can be directed to the corresponding authors.

## Supplementary Materials

The following supporting information can be downloaded at: https://www.mdpi.com/article/10.3390/jof12040261/s1, Figure S1: Photographs of *T. fuciformis* fruiting bodies cultivated on different substrate formulations; Figure S2: Analysis of the top 20 major metabolites in *T. fuciformis* cultivated on different substrates; Figure S3: PCA of untargeted metabolomics in *T. fuciformis* cultivated on different substrates; Figure S4: OPLS-DA analysis of untargeted metabolomics in *T. fuciformis* cultivated on different substrates; Figure S5: Relative content and differential metabolite numbers of amino acids and derivatives, saccharides, and flavoring substances in *T. fuciformis* cultivated on different substrates; Figure S6: KEGG enrichment analysis of the top 20 differential metabolites in *T. fuciformis* cultivated on different substrates; Figure S7: Comparison of polysaccharide levels in spores isolated from *T. fuciformis* cultivated on different substrates. Table S1: Cultivation formulations of *T. fuciformis* on different substrates.
