# Supplementary material for: Effects of Cultivation Substrate Differences on Quality Formation and Polysaccharide Composition Characteristics of Tremella fuciformis
Source: J Fungi (Basel). 2026 Apr 3;12(4):261. doi: 10.3390/jof12040261 (PMC13117257; doi:10.3390/jof12040261)
Supplement: Supplementary file 1 [file jof-12-00261-s001.zip › jof-4189130-supplementary.pdf]

Supplementary materials

Effects of Cultivation Substrate Differences on Quality Formation and Polysaccharide Composition Characteristics of *Tremella fuciformis*

Jianqiu Chen [1,†], Yating Deng [1,†], Yujie Chen [1], Keming Zhu [1], Xun Yao [1], Shenqiao Yang [1], Liding Chen [1,2,3*], Shujing Sun [1,2,3*]

[1] College of Life Sciences, Fujian Agriculture and Forestry University, Fuzhou 350002, China
[2] Gutian Edible Fungi Research Institute, Fujian Agriculture and Forestry University, Ningde 352200, China
[3] Fujian Edible Fungi Industry Technology Innovation Research Institute, Fuzhou 350002, China
[*] Correspondence: chenliding@fafu.edu.cn, shjsun2004@126.com
[†] These authors contributed equally to this work

Figures

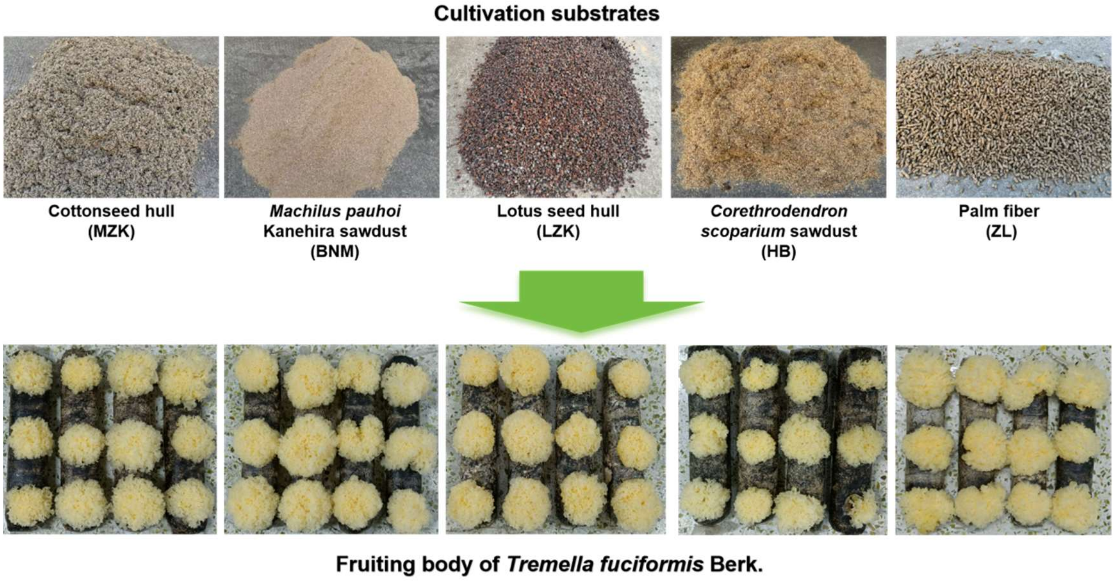

Figure S1. Photographs of *T. fuciformis* fruiting bodies cultivated on different substrate formulations.

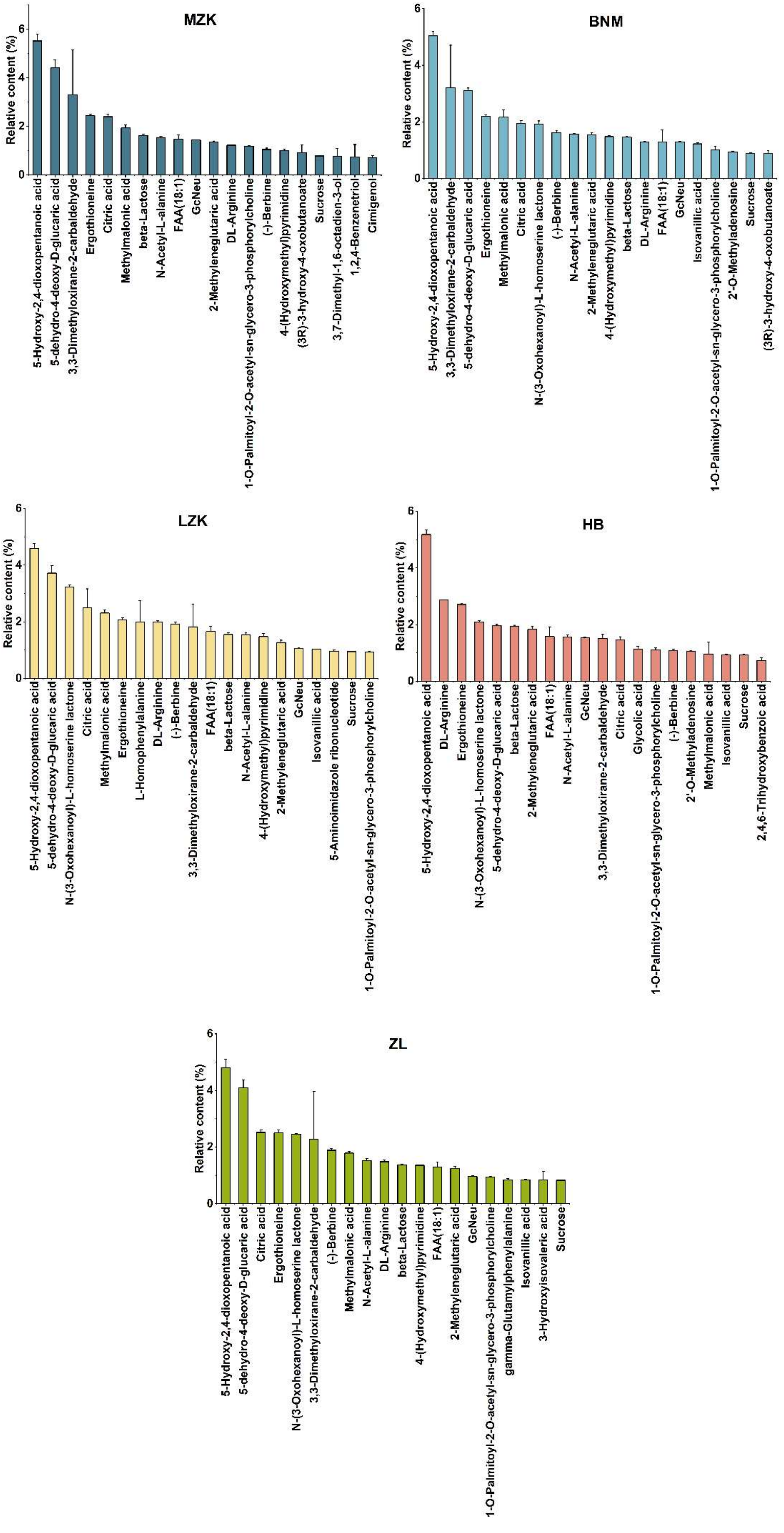
MZK
Relative content (%)
0
2
4
6
5-Hydroxy-2,4-dioxopentanoic acid
5-dehydro-4-deoxy-D-glucaric acid
3,3-Dimethyloxirane-2-carbaldehyde
Ergothioneine
Citric acid
Methylmalonic acid
beta-Lactose
N-Acetyl-L-alanine
FAA(18:1)
GcNeu
2-Methyleneglutaric acid
DL-Arginine
1-O-Palmitoyl-2-O-acetyl-sn-glycero-3-phosphorylcholine
(-)-Berbine
4-(Hydroxymethyl)pyrimidine
(3R)-3-hydroxy-4-oxobutanoate
Sucrose
3,7-Dimethyl-1,6-octadien-3-ol
1,2,4-Benzenetriol
Cimigenol
BNM
Relative content (%)
0
2
4
6
5-Hydroxy-2,4-dioxopentanoic acid
3,3-Dimethyloxirane-2-carbaldehyde
5-dehydro-4-deoxy-D-glucaric acid
Ergothioneine
Methylmalonic acid
Citric acid
N-(3-Oxohexanoyl)-L-homoserine lactone
(-)-Berbine
N-Acetyl-L-alanine
2-Methyleneglutaric acid
4-(Hydroxymethyl)pyrimidine
beta-Lactose
DL-Arginine
FAA(18:1)
GcNeu
Isovanillic acid
1-O-Palmitoyl-2-O-acetyl-sn-glycero-3-phosphorylcholine
2'-O-Methyladenosine
Sucrose
(3R)-3-hydroxy-4-oxobutanoate
LZK
Relative content (%)
0
2
4
6
5-Hydroxy-2,4-dioxopentanoic acid
5-dehydro-4-deoxy-D-glucaric acid
N-(3-Oxohexanoyl)-L-homoserine lactone
Citric acid
Methylmalonic acid
Ergothioneine
L-Homophenylalanine
DL-Arginine
(-)-Berbine
3,3-Dimethyloxirane-2-carbaldehyde
FAA(18:1)
beta-Lactose
N-Acetyl-L-alanine
4-(Hydroxymethyl)pyrimidine
2-Methyleneglutaric acid
GcNeu
Isovanillic acid
5-Aminoimidazole ribonucleotide
Sucrose
1-O-Palmitoyl-2-O-acetyl-sn-glycero-3-phosphorylcholine
HB
Relative content (%)
0
2
4
6
5-Hydroxy-2,4-dioxopentanoic acid
DL-Arginine
Ergothioneine
N-(3-Oxohexanoyl)-L-homoserine lactone
5-dehydro-4-deoxy-D-glucaric acid
beta-Lactose
2-Methyleneglutaric acid
FAA(18:1)
N-Acetyl-L-alanine
GcNeu
3,3-Dimethyloxirane-2-carbaldehyde
Citric acid
Glycolic acid
1-O-Palmitoyl-2-O-acetyl-sn-glycero-3-phosphorylcholine
(-)-Berbine
2'-O-Methyladenosine
Methylmalonic acid
Isovanillic acid
Sucrose
2,4,6-Trihydroxybenzoic acid
ZL
Relative content (%)
0
2
4
6
5-Hydroxy-2,4-dioxopentanoic acid
5-dehydro-4-deoxy-D-glucaric acid
Citric acid
Ergothioneine
N-(3-Oxohexanoyl)-L-homoserine lactone
3,3-Dimethyloxirane-2-carbaldehyde
(-)-Berbine
Methylmalonic acid
N-Acetyl-L-alanine
DL-Arginine
beta-Lactose
4-(Hydroxymethyl)pyrimidine
FAA(18:1)
2-Methyleneglutaric acid
GcNeu
1-O-Palmitoyl-2-O-acetyl-sn-glycero-3-phosphorylcholine
gamma-Glutamylphenylalanine
Isovanillic acid
3-Hydroxyisovaleric acid
Sucrose

Figure S2. Analysis of the top 20 major metabolites in *T. fuciformis* cultivated on different substrates.

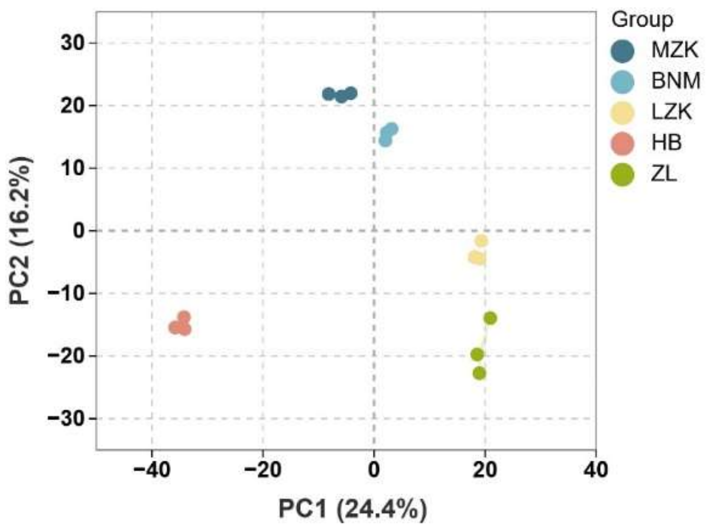

Figure S3. PCA analysis of untargeted metabolomics in *T. fuciformis* cultivated on different substrates.

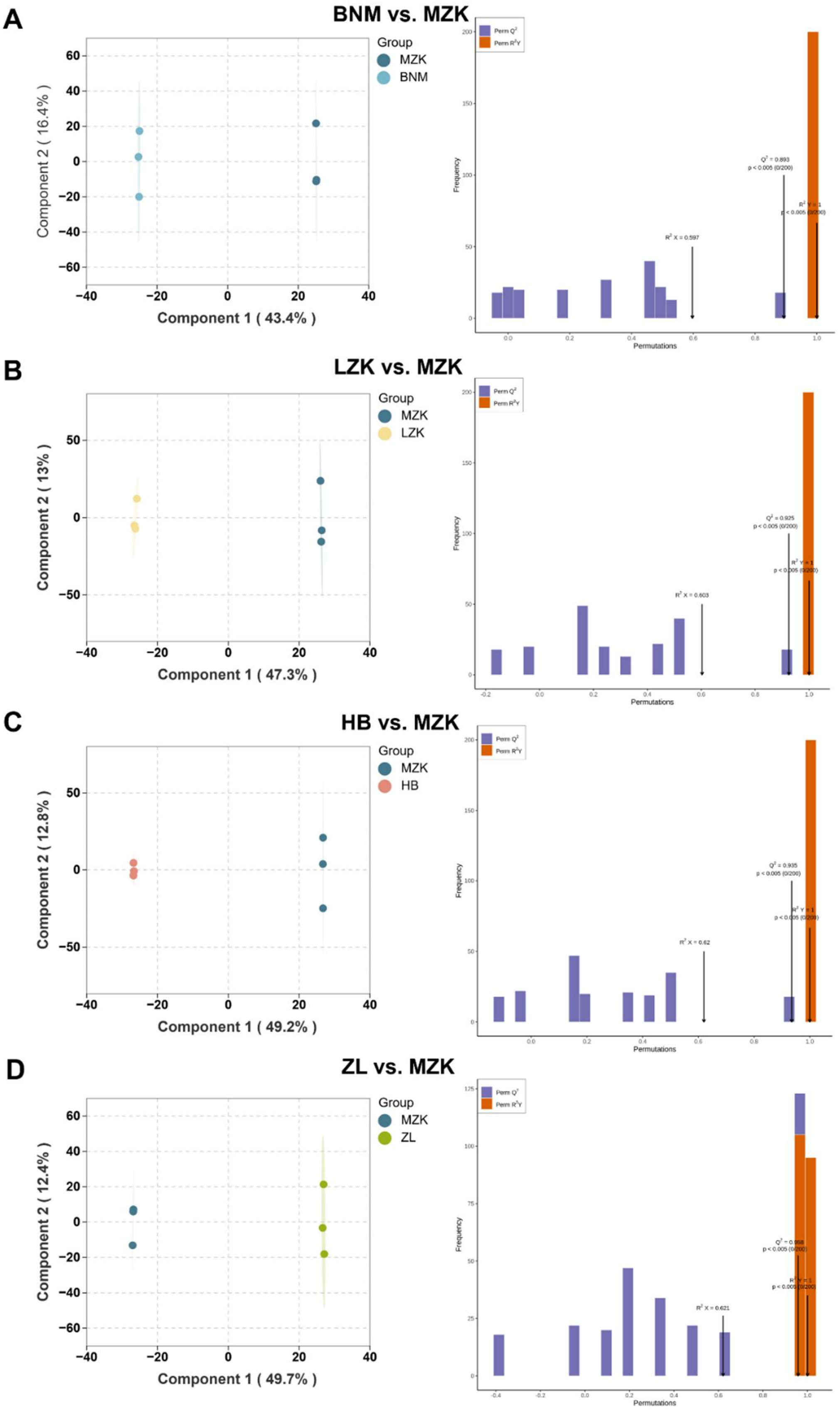

Figure S4. OPLS-DA analysis of untargeted metabolomics in *T. fuciformis* cultivated on different substrates.

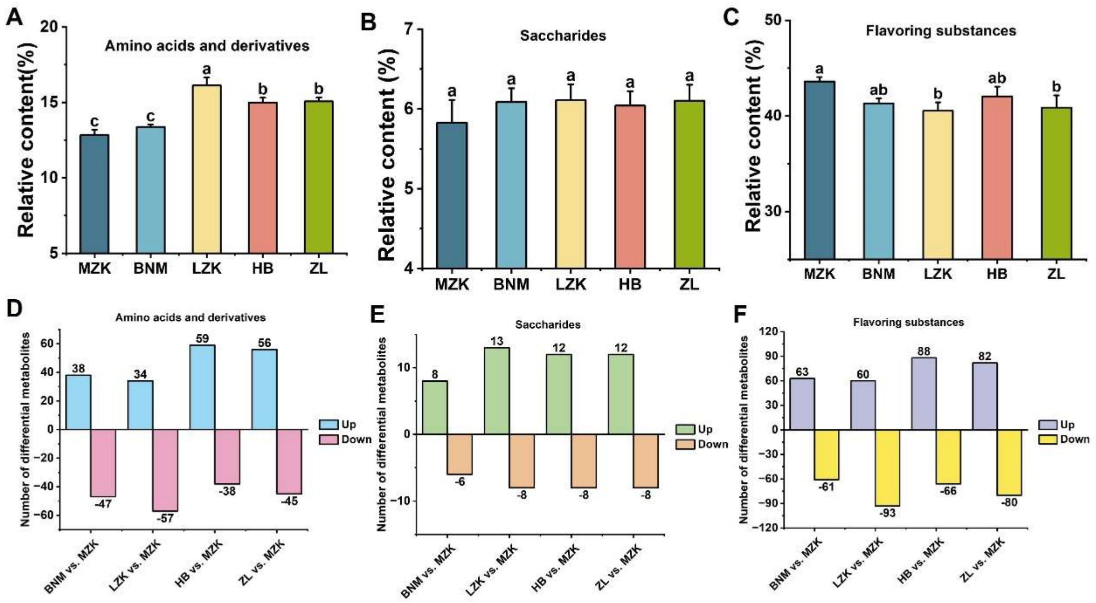

Figure S5. Relative content and differential metabolite numbers of amino acids and derivatives, saccharides, and flavoring substances in *T. fuciformis* cultivated on different substrates. (A) Relative content of amino acids and derivatives, (B) Relative content of saccharides, (C) Relative content of flavoring substances, (D) Number of differential amino acids and derivatives, (E) Number of differential saccharides, (F) Number of differential flavoring substances.

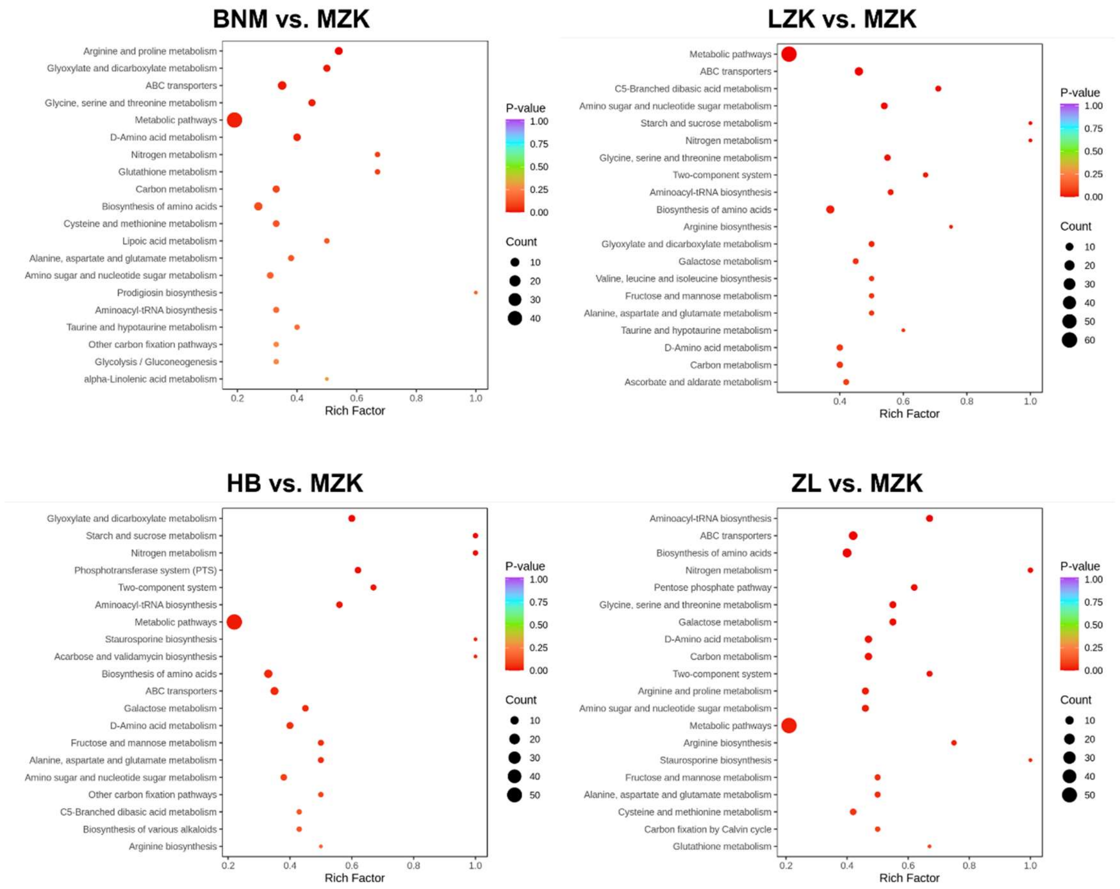

Figure S6. KEGG enrichment analysis of the top 20 differential metabolites in *T. fuciformis* cultivated on different substrates.

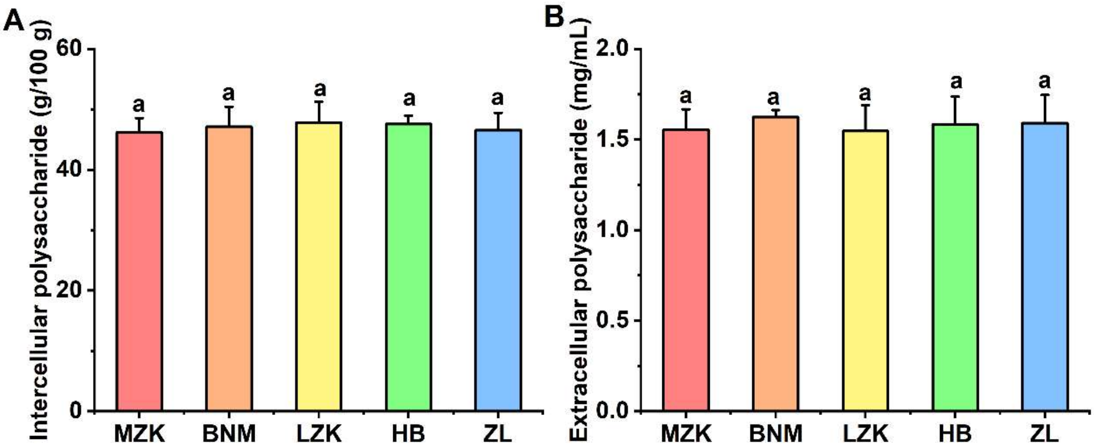

Figure S7. Comparison of polysaccharide levels in spores isolated from *T. fuciformis* cultivated on different substrates (A) Intracellular polysaccharides, (B) Extracellular polysaccharides. Different lowercase letters indicate significant differences at the $P < 0.05$ level; n = 3 for each group.

Tables

Table S1. Cultivation formulations of *T. fuciformis* on different substrates.

Variety	Formulations	Primary Ingredient	Primary Ingredient (%)	Cottonseed hulls (%)	Wheat bran (%)	Gypsum (%)
TYH-SD1	Formulation1	Cottonseed hulls (MZK)	55	20	24	1
	Formulation2	*Machilus pauhoi* Kanehira sawdust (BNM)				
	Formulation3	Lotus seed hulls (LZK)				
	Formulation4	*Corethrodendron scoparium* sawdust (HB)				
	Formulation5	Palm fiber (ZL)				
